# Dissociated leg muscle atrophy in amyotrophic lateral sclerosis/motor neuron disease: the ‘split-leg’ sign

**DOI:** 10.1038/s41598-020-72887-7

**Published:** 2020-09-24

**Authors:** Young Gi Min, Seok-Jin Choi, Yoon-Ho Hong, Sung-Min Kim, Je-Young Shin, Jung-Joon Sung

**Affiliations:** 1 Department of Neurology, Seoul National University Hospital, Seoul National University College of Medicine, 101 Daehak-ro, Jongno-gu, Seoul, 03080 Republic of Korea; 2 Department of Neurology, Inha University Hospital, Incheon, Republic of Korea; 3 Department of Neurology, Seoul Metropolitan Government Seoul National University Boramae Medical Center, Seoul, Republic of Korea

**Keywords:** Motor neuron disease, Neurodegenerative diseases

## Abstract

Disproportionate muscle atrophy is a distinct phenomenon in amyotrophic lateral sclerosis (ALS); however, preferentially affected leg muscles remain unknown. We aimed to identify this split-leg phenomenon in ALS and determine its pathophysiology. Patients with ALS (n = 143), progressive muscular atrophy (PMA, n = 36), and age-matched healthy controls (HC, n = 53) were retrospectively identified from our motor neuron disease registry. We analyzed their disease duration, onset region, ALS Functional Rating Scale-Revised Scores, and results of neurological examination. Compound muscle action potential (CMAP) of the extensor digitorum brevis (EDB), abductor hallucis (AH), and tibialis anterior (TA) were reviewed. Defined by CMAP_EDB_/CMAP_AH_ (SI_EDB_) and CMAP_TA_/CMAP_AH_ (SI_TA_), respectively, the values of split-leg indices (SI) were compared between these groups. SI_EDB_ was significantly reduced in ALS (p < 0.0001) and PMA (p < 0.0001) compared to the healthy controls (HCs). SI_TA_ reduction was more prominent in PMA (p < 0.05 vs. ALS, p < 0.01 vs. HC), but was not significant in ALS compared to the HCs. SI was found to be significantly decreased with clinical lower motor neuron signs (SI_EDB_), while was rather increased with clinical upper motor neuron signs (SI_TA_). Compared to the AH, TA and EDB are more severely affected in ALS and PMA patients. Our findings help to elucidate the pathophysiology of split-leg phenomenon.

## Introduction

Amyotrophic lateral sclerosis (ALS) is a catastrophic neurodegenerative disorder affecting the upper motor neurons (UMNs) and lower motor neurons (LMNs), characterized by a relentless progression of skeletal muscle weakness. The onset region, rate of progression, and relative dominance of UMN or LMN degeneration widely vary among patients with ALS. Unfortunately, there has been no reliable diagnostic biomarker for ALS, and so the early diagnosis is often difficult, especially in those presenting with leg weakness^1,2^.

“Split-hand” syndrome is a well-characterized ALS-specific phenomenon that refers to preferential wasting of thenar muscles, including the first dorsal interosseous (FDI) and the abductor pollicis brevis (APB), compared to hypothenar muscles, including the abductor digiti minimi (ADM)^3–8^. Because FDI and ADM are innervated by the same motor nerve and share an identical myotome, this phenomenon may imply a cortical bias; the larger somatotopic representation for thenar muscles may lead to differential degeneration of anterior horn cells via glutamate excitotoxicity^9–14^. This idea has been supported by several transcranial magnetic stimulation (TMS) studies. On the contrary, a few studies suggested primary dysfunction of spinal motor neurons or peripheral axons^6,15,16^.

Recently, the “split-leg” sign in ALS was introduced as preferential wasting of gastrocnemius (ankle plantar flexor) compared to tibialis anterior (TA; ankle dorsiflexor)^17^. However, this contrasts to clinical experience in which ALS patients sometimes present with foot drop^18^. Of relevance, two successive studies that compared extensor digitorum brevis (EDB) and abductor hallucis (AH) muscles proposed larger degree of atrophy in the former^19,20^. Nonetheless, these studies did not compare the leg and foot muscles simultaneously nor investigate other forms of motor neuron disease such as progressive muscular atrophy and flail leg syndrome. Moreover, only one study included an ALS mimic (lumbar spondylosis) in its analysis, whereas the others compared ALS patients to healthy controls^20^. In the present study, we compared compound muscle action potentials (CMAPs) of EDB, AH, and TA in ALS, progressive muscular atrophy (PMA), which is characterized solely by signs of LMN degeneration, and age-matched healthy controls (HC). If selective atrophy is present in ALS, we questioned whether it is also present in PMA. Further, through correlating clinical variables with the split-leg index (SI), we tried to gain insight into the underlying pathophysiology.

## Results

### Clinical characteristics

Table 1 summarizes the demographic and clinical characteristics of the three groups. The number of eligible subjects was 143, 36, and 53 in the ALS, PMA, and HC groups, respectively. The age distribution was similar between groups (ALS: 61.57 ± 10.74, PMA: 59.92 ± 10.81, HC: 62.98 ± 8.17 years). The female to male ratio was significantly lower in the PMA group compared to the other groups (ALS: 1.1, PMA: 0.2, HC: 1.4, p < 0.0001). Between ALS and PMA groups, there was no significant difference in the frequency of onset regions, disease duration, or total ALS Functional Rating Scale-Revised (ALSFRS-R) and subALSFRS-R scores. Based on neurological examinations, clinical UMN signs were present in 91% of ALS patients, while no patients in the PMA group were found to have UMN signs. Clinical LMN signs were found in 76% and 81% of ALS and PMA patients, respectively.

**Table 1:** Baseline clinical and electrophysiologic characteristics of the participants in each group.

Variables	ALS	PMA	HC	Statistic difference
Number of patients	143	36	53	N/A
Age	61.57 ± 10.74	59.92 ± 10.81	62.98 ± 8.17	NS
Gender (female:male)	1.1 (75:68)	0.2 (6:30)	1.4 (31:22)	PMA-ALS: p < 0.001 PMA-HC: p < 0.001
Onset region (B/C/LS)	45/48/50	8/13/15	N/A	NS
Disease duration	19.20 ± 19.73	22.44 ± 25.63	N/A	NS
ALSFRS-R score	36.47 ± 6.25	36.88 ± 5.74	N/A	NS
subALSFRS-R score (for items 7–9)	7.46 ± 2.96	7.26 ± 3.43	N/A	NS
Clinical UMN sign	130 (91%)	0 (0%)	N/A	N/A
Clinical LMN sign	109 (76%)	29 (81%)	N/A	N/A

*ALS* amyotrophic lateral sclerosis, *PMA* progressive muscular atrophy, *HC* healthy control, *N/A* not applicable, *NS* not significant, *B* bulbar, *C* cervical, *LS* lumbosacral, *ALSFRS-R* revised amyotrophic lateral sclerosis functional rating scale, *UMN* upper motor neuron, *LMN* lower motor neuron.

### Electrophysiological data

We analyzed 171, 40, and 79 legs in the ALS, PMA, and HC groups, respectively. Among them, CMAP_TA_ was obtained in 119 (70%), 26 (65%), and 14 (18%) legs in the ALS, PMA, and HC groups, respectively. CMAP_EDB_, CMAP_TA_, and CMAP_AH_ amplitudes were much smaller in ALS and PMA groups than those in the HC group (CMAP_EDB_ (mV): ALS 2.80 [0.85–5.10], PMA 2.35 [0.57–5.22], HC 6.60 [4.95–8.70], CMAP_TA_: ALS 6.20 [2.85–8.70], PMA 3.55 [1.60–6.47], HC 11.15 [10.18–14.32], CMAP_AH_: ALS 15.20 [6.40–20.95], PMA 17.20 [11.25–22.43], HC 22.30 [17.75–26.00], p < 0.0001 for all comparisons) (Supplementary Fig. 1). CMAP was not elicited in the following legs: CMAP_EDB_/CMAP_TA_/CMAP_AH_; 29/7/5 in the ALS group, 6/2/1 in the PMA group, and none in the HC group. Limbs with no elicitable CMAP_AH_ were excluded from the calculation of SI, as it was defined as the denominator.

**Figure 1:**
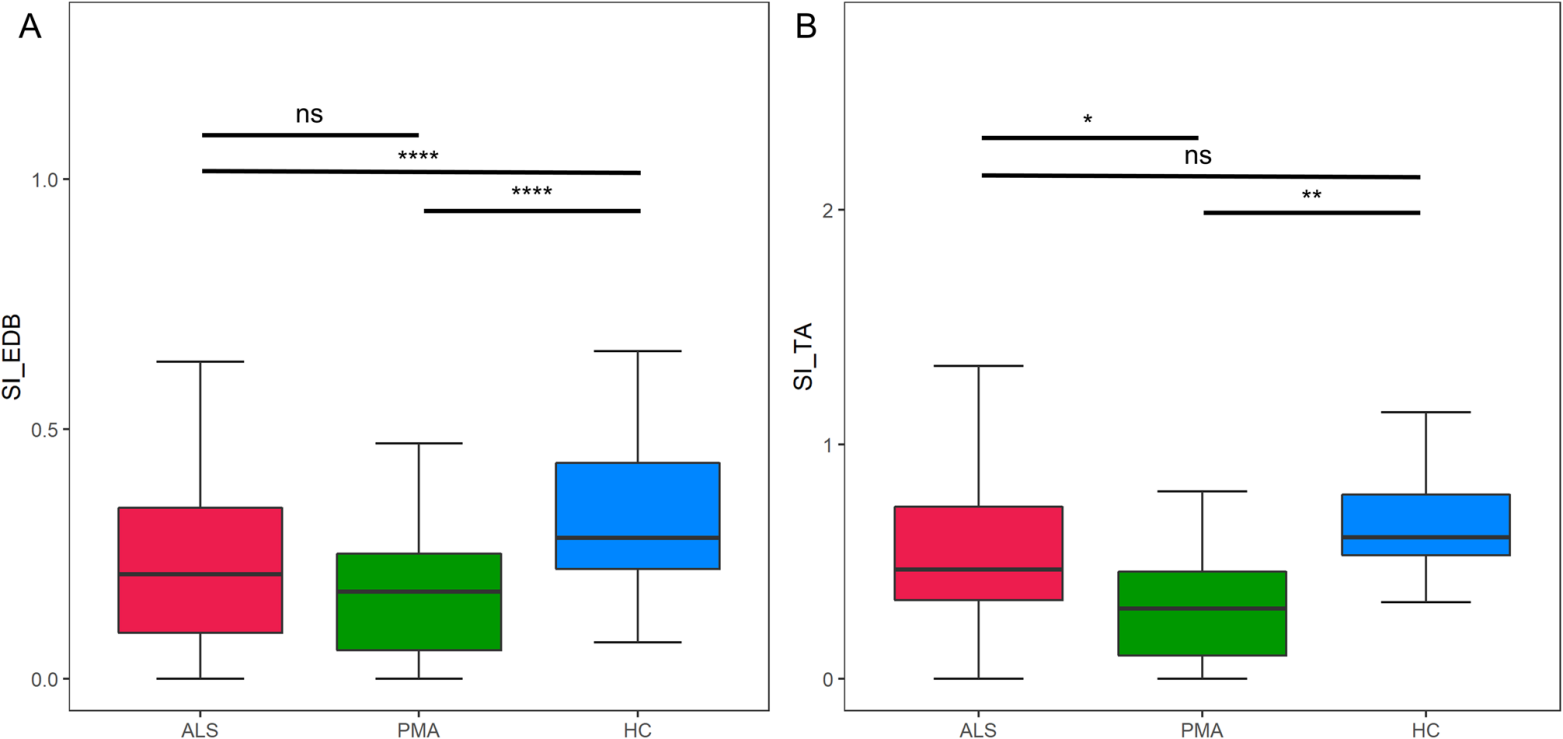
Split-leg index (SI_EDB_, SI_TA_) according to the diagnosis. *p < 0.05, **p < 0.01, ****p < 0.0001. ALS and PMA showed significant reduction in SI_EDB_ compared to HC (**A**). Reduction in SI_TA_ was also observed in PMA but was not significant in ALS compared to HC (**B**).

The SI in each group is represented using box plots in Fig. 1. Compared to HCs, SI_EDB_ was significantly lower in ALS and PMA groups, while it was comparable between the two diseased groups (ALS 0.21 [0.09–0.34], PMA 0.17 [0.06–0.25], HC 0.31 [0.22–0.43]). The SI_TA_ of the PMA group was significantly lower than those of the ALS and HC groups (p < 0.05 vs. ALS, p < 0.01 vs. HC). SI_TA_ was lower in the ALS group compared to HCs, but the difference did not reach statistical significance (ALS 0.47 [0.34–0.74], PMA 0.30 [0.10–0.46], HC 0.60 [0.53–0.79]).

**Figure 2:**
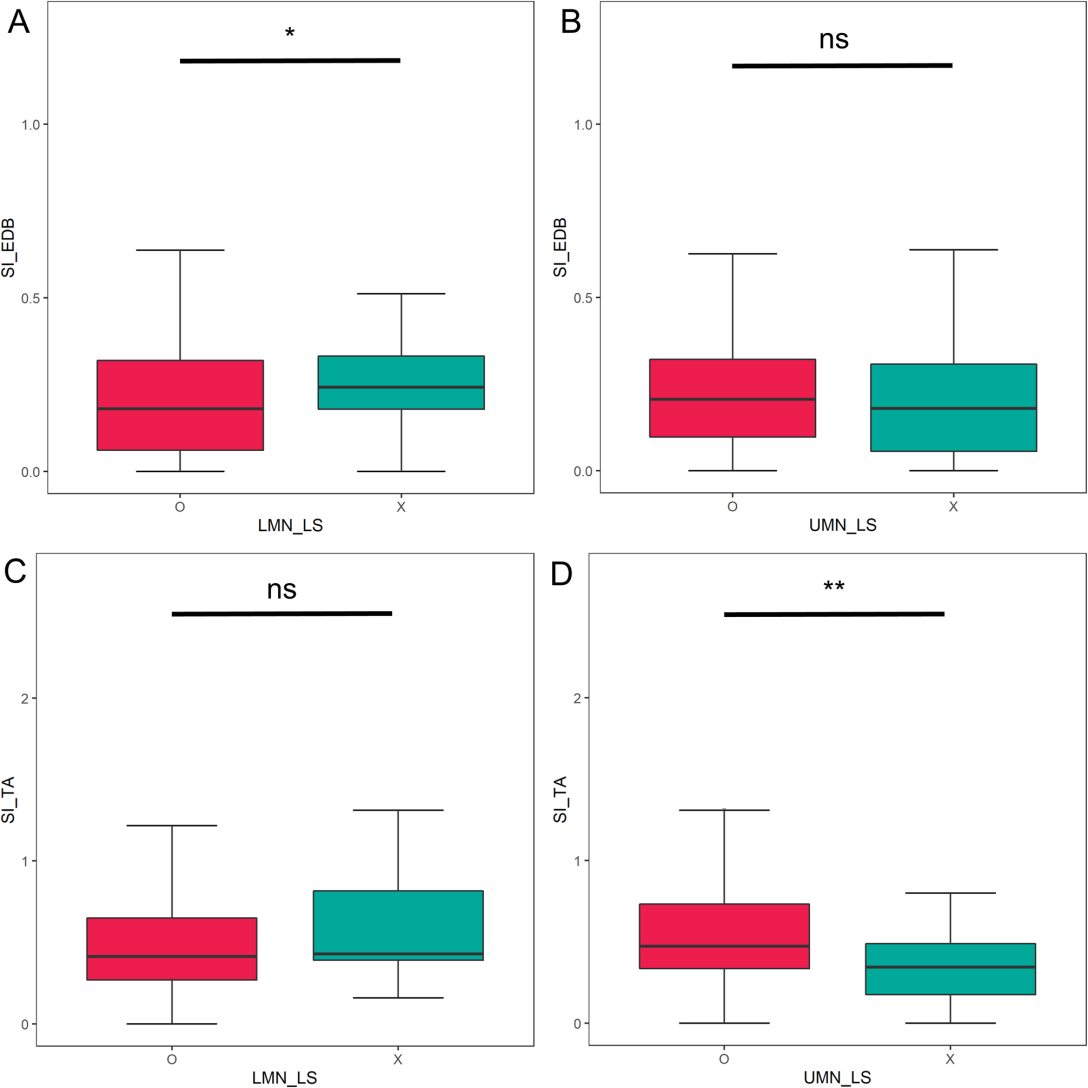
Correlation between split-leg index and the presence of lumbosacral upper/lower motor neuron sign in MND patients. *p < 0.05, **p < 0.01. SI_EDB_ was significantly decreased with the positive LMN sign (p < 0.05) (**A**). The presence of UMN sign was associated with increased SI_TA_ (p < 0.05) (**D**). Although other comparisons were not statistically significant, SI tended to be lower with LMN sign (**C**) and higher with UMN sign (**B**).

**Figure 3:**
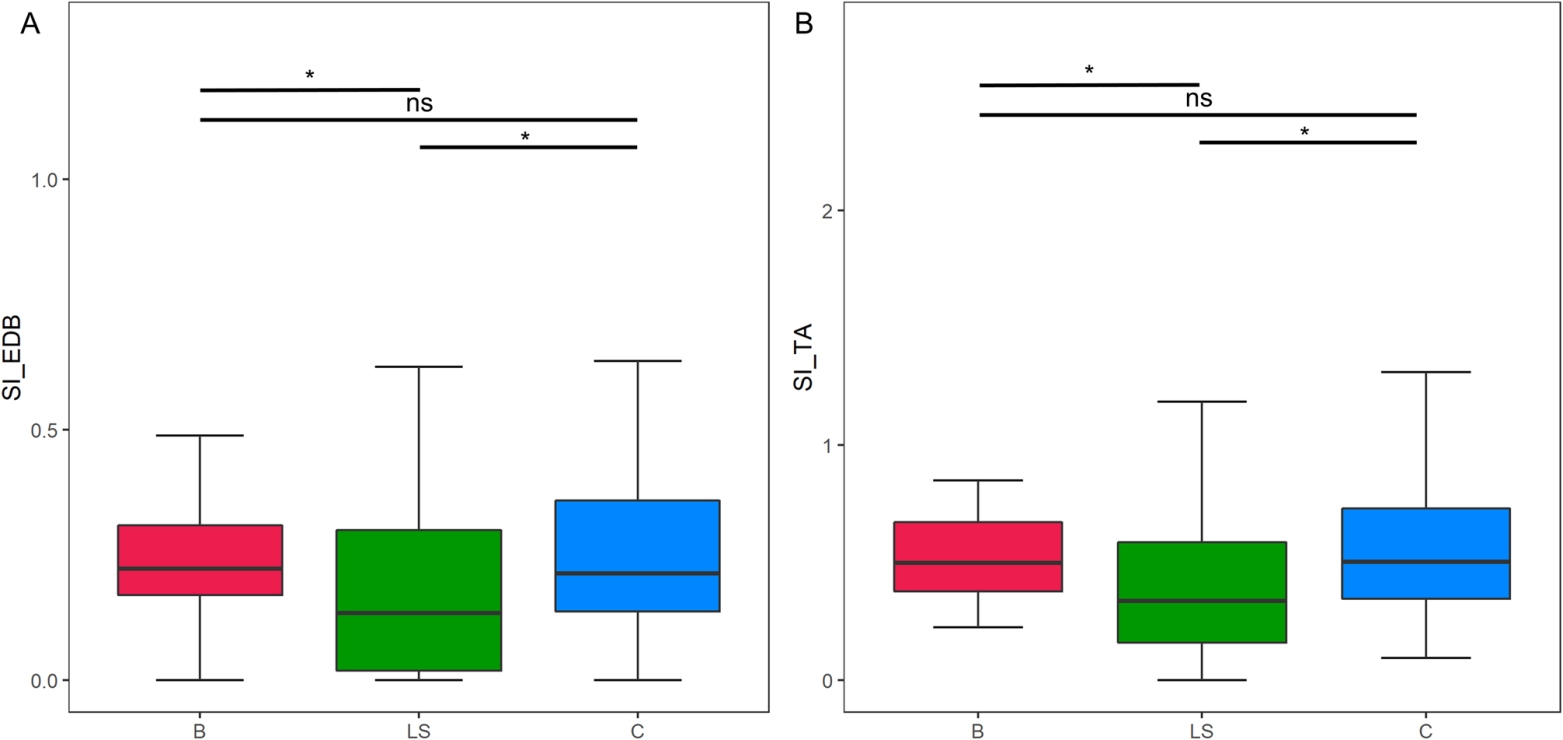
Distribution of split-leg index according to the onset region. *p < 0.05. In both SI_EDB_ and SI_TA_, significant reduction in lower-limb-onset MND group (LS) was found compared to the others (**B**,**C**).

To investigate whether clinical variables affected dissociated leg muscle atrophy, we compared the SI of motor neuron disease (MND; ALS or PMA) legs according to the clinical UMN or LMN signs in the lumbosacral segment (Fig. 2). Both SI_EDB_ and SI_TA_ were modestly lower in legs with positive LMN signs, but statistical significance (p < 0.05) was only met in the comparison of SI_EDB_. SI_TA_ was significantly increased in legs with positive UMN signs (p < 0.01). When we correlated SI with the site of disease onset, both SIs were significantly lower in lower-limb-onset MND compared to bulbar or upper-limb-onset MND (SI_EDB_: bulbar-onset 0.22 [0.17–0.31], upper-limb-onset 0.21 [0.14–0.36], lower-limb-onset 0.13 [0.02–0.30]. SI_TA_: bulbar-onset 0.50 [0.38–0.67], upper-limb-onset 0.50 [0.34–0.73], lower-limb-onset 0.34 [0.16–0.59], p < 0.05 for lower-limb-onset versus bulbar or upper-limb-onset groups, both SI_EDB_ and SI_TA_) (Fig. 3).

**Figure 4:**
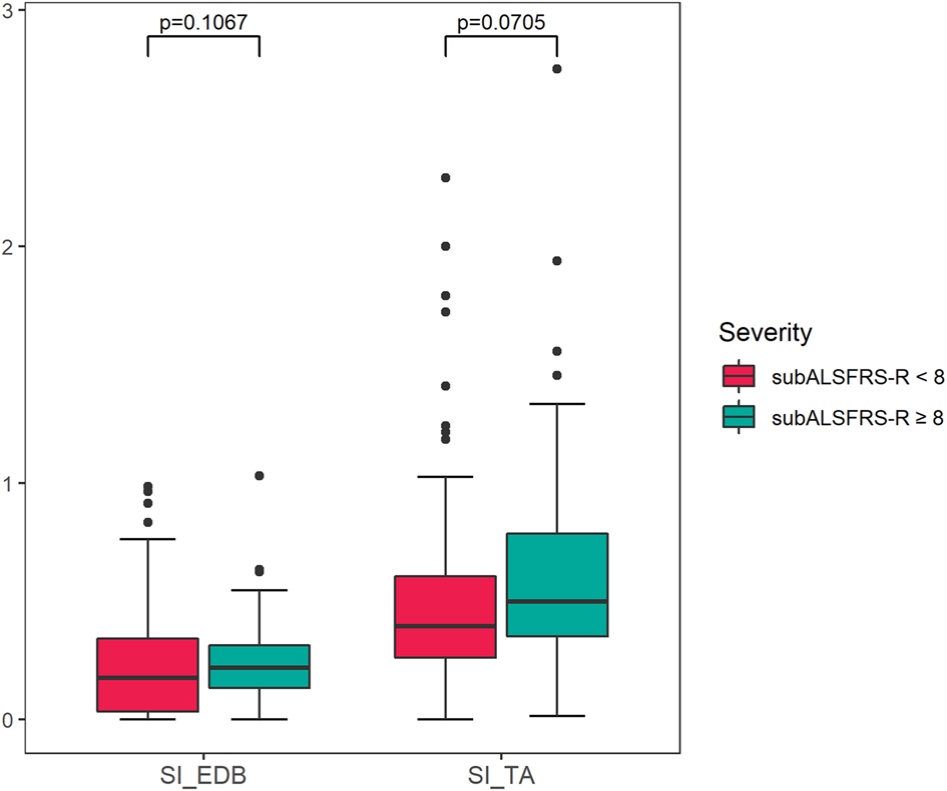
Split-leg index according to subALSFRS-R score of MND patients. Both SI_EDB_ and SI_TA_ tended to be decreased with clinical severity, represented by subALSFRS-R score < 8.

Next, we divided MND legs into two groups based on the subALSFRS-R score (median score: 8) to correlate SI with the severity of lower limb dysfunction. Although it did not reach statistical significance, the severe group (n = 100, subALSFRS-R < 8) showed a lower SI_EDB_ as well as SI_TA_ than the mild group (n = 101, subALSFRS-R ≥ 8) (SI_EDB_: severe 0.17 [0.03–0.34] vs. mild 0.22 [0.13–0.31], p = 0.11; SI_TA_: severe 0.39 [0.26–0.61] vs. mild 0.50 [0.35–0.79], p = 0.07) (Fig. 4).

## Discussion

Our study showed preferential degeneration of EDB and TA compared to AH, a great toe flexor/abductor. While EDB extends the toes in conjunction with TA-mediated ankle dorsiflexion, AH is usually co-activated during plantar flexion of the ankle. Thus, we propose that dorsiflexors of lower limbs in ALS are more heavily affected than plantar flexors. This is in concordance with previous clinical observations that patients with ALS commonly present with foot drop^17–19^. Notably, the so-called split-leg phenomenon was also present and was even more prominent in PMA.

To date, cortical influence is the dominant theory explaining unbalanced muscle involvement in ALS. Denser corticospinal input to thenar muscles that facilitates precise hand motions, unique to humans, might lead to premature degeneration. Accordingly, previous studies on split-leg syndrome have suggested a cortical mechanism^17,18,20^. A human functional MRI during ankle dorsiflexion/plantar flexion revealed broader cortical representation for dorsiflexion^21^. Another study using TMS showed exclusive short-interval facilitation in TA compared to gastrocnemius and soleus^22^. The above findings indicate that ankle dorsiflexors receive a greater innervation from corticospinal neurons, which also correlates with our results. However, in these studies, EDB and AH were not specifically investigated. Indeed, corticospinal input might considerably differ, even between the nearest muscles. Thus, further analyses are necessary to fully understand UMN innervations of the lower-limb musculature and to confirm the corticofugal mechanism of split-leg phenomenon^23–26^.

Of note, cortical influences do not fully explain our findings. Patients with PMA, who did not show any clinical UMN signs, revealed prominent split-leg signs. Moreover, SI_EDB_ in MND patients with clinical LMN signs was significantly lower than that in those without. Accordingly, we propose additional peripheral pathophysiological mechanisms to support these results. In ALS, it is known that lower motor neurons innervating fast-twitching myofibers, with larger soma and higher innervation ratios, are especially prone to degeneration^27^. Hence, heterogeneous anterior horn cell-composition within lower limbs might attribute to the uneven atrophy. In addition, repeated phasic activation of dorsiflexors during locomotion might produce greater oxidative stress in these antigravity muscles, leading to premature dieback^28,29^.

Another interesting finding in our study was an increased split-leg index (SI_TA_) in the presence of clinical UMN signs. Considering the decreased SI in ALS, this was an unexpected result. A higher SI with definite UMN signs might be possibly caused by the inclusion of PMA patients with lower SI in the no-UMN-sign group. Additionally, spinal interneurons or Renshaw cells, which mediate recurrent inhibition of spinal motoneurons exhibit an altered physiologic status in ALS^30^. Increased potentiation of the descending medial longitudinal fasciculus following corticospinal tract lesions was reported^31^. Pathologically, the presence of TDP-43 pathology in these tracts have been reported in some ALS autopsies^32,33^. These anterior horn cell-modulators may be involved in this “inverse” split-leg phenomenon.

In group comparisons according the onset region, SI was the lowest in patients with lower-limb onset. The disease duration for each patient group was as follows: bulbar 12 [7–16.25], upper-limb 15^8,22^, lower-limb 15 [10–24.75], p < 0.05. Therefore, the degeneration of lumbosacral spinal motor neurons is estimated to have started earliest in patients with lower-limb onset, given the modestly longer or at least comparable disease duration. Moreover, SI was slightly lower in patients with severe functional disability in their legs (subALSFRS-R score $\ge$ 8), although there was no statistical significance. These results may possibly indicate that SI continues to decrease along with disease progression. To investigate the temporal changes of SI in the course of disease, further prospective studies with follow-up measurements of SI are warranted.

It can be argued that PMA should not be regarded as a lower motor neuron disease since profound LMN degeneration might mask clinical UMN signs. Autopsy studies have shown frequent subclinical involvement of the corticospinal tract in PMA^34^. At the very least, PMA could be considered an LMN-predominant ALS. In our study, the disease severity represented by the subALSFRS-R score was comparable across ALS and PMA groups. Therefore, we speculated that the lower SI in PMA is not due to the degeneration of pyramidal tract, but rather to the peripheral compartment. Consistent with previous epidemiological studies, the proportion of males in PMA far exceeded that in those with ALS or HCs^35^. Thus, we investigated whether sex physiologically affected the relative ratio of CMAP_EDB_ and CMAP_AH_. As a result, an even higher SI was observed in males (p < 0.001, Supplementary Fig. 2), which suggests that the difference in sex ratio may not have confounded our results.

We acknowledge several limitations in our study. (1) Due to incomplete medical records, we could not collect motor grades of each muscle. Nevertheless, the fact that dorsiflexion is more severely affected than plantar flexion is readily agreed upon in previous studies and matches with our electrophysiologic observations. (2) The nerve conduction study (NCS) of the TA was not conducted on every occasion, but when the NCS of the EDB produced any abnormalities on CMAP, nerve conduction velocity, or F-waves, the TA was studied. As a result, CMAP_TA_ was obtained from 119/171 (70%), 26/40 (65%), 14/79 (18%) lower limbs of ALS, PMA, HC participants, respectively. Therefore, our results of SI_TA_ might have been biased and underpowered. (3) In some patients, the NCS had been conducted on bilateral legs of a single subject (ALS 28/143 [19.5%], PMA 4/36 [11%], HC 26/53 [49%]). These data were included as separate legs in the analysis of the SI. In contrast, clinical information including UMN/LMN signs and disease duration had not been recorded separately, thus regarded identical. ALSFRS-R scores did not reflect potential asymmetry in the same manner. Therefore, our clinico-electrophysiologic correlation may have been biased. However, the proportion of MND patients with the NCS performed bilaterally was minimal (ALS: 19.5%, PMA: 11%), and none of them showed marked clinical asymmetry. (4) CMAPs measure the number of excitable muscle fibers in a given muscle, not reliably estimating the number of surviving motor units when assessing the rate and distribution of disease progression. Motor unit number estimation (MUNE) techniques, which have been developed for these reasons, can be applied in a longitudinal study for split-leg phenomenon, although it is not suitable for cross sectional studies. (5) The lack of detailed electrophysiologic investigations due to the retrospective nature of the study limits the mechanistic implications of our study; MUNE studies, TMS, F-wave, and H-reflex (for example, F-wave persistence or H_max_/M_max_) were not investigated.

The current study showed that TA and EDB, muscles activated during ankle dorsiflexion, are preferentially wasted compared to AH. Interestingly, the split-leg sign was even more pronounced in PMA patients. This finding is not solely explained by a cortical mechanism, thus implies the contribution of a peripheral mechanism. As such, selective vulnerability of alpha motor neurons or myofiber pools might be a possible explanation. Neuromuscular constituents of lower limbs require further investigation in terms of their relative metabolic and physiologic changes in ALS.

## Methods

### Subjects

From the MND registry of Seoul National University Hospital, 218 ALS and 41 PMA patients were identified between January 2017 and December 2019. We included patients whose lower limbs were affected by the disease, irrespective of the onset region. An affected leg was defined by one of the following: (1) objective weakness in a neurological examination, (2) any point loss in ALSFRS-R subscore for items 7$-$9, or (3) both denervation and reinnervation potentials on needle electromyography (EMG).

Based on the revised El Escorial criteria, patients with definite or probable ALS (n = 164) were included in the study^36^. Patients were excluded if the NCS was not performed within three months of the clinical assessment (n = 13). Patients with concurrent polyneuropathy were excluded (n = 6). Two patients were excluded because their legs were not affected. If the NCS was performed in bilateral legs in one subject, the two sets of data were both included. As a result, 171 symptomatic legs from 143 ALS patients were included in the analysis. A diagnosis of PMA was determined by the presence of both clinical and electrophysiological LMN signs (denervation and reinnervation on EMG) in more than two segments without evidence of UMN signs. Clinical UMN signs did not develop during the follow-up period of more than 1 year in all patients. The exclusion criteria included not having NCS data (n = 2), concurrent polyneuropathy (n = 2), and unaffected legs (n = 1). As a result, 40 affected legs from 36 PMA patients were included in the analysis.

**Figure 5:**
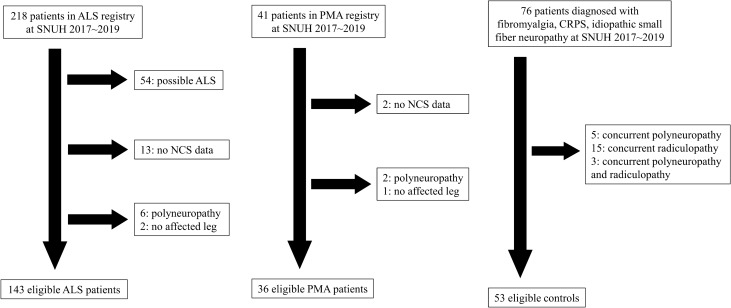
Flow chart for the selection of eligible patients. All figures were created by YGM using Microsoft PowerPoint (https://products.office.com/en-in/powerpoint version 16.16.3 (181015)).

For age-matched controls, we screened 76 patients aged > 50 years, having small fiber neuropathy, fibromyalgia, or complex regional pain syndrome as a presumptive diagnosis in the same period (from January 2017 to December 2019). Those co-affected by lumbosacral radiculopathy or peripheral neuropathy were excluded (n = 23). As a result, 79 unaffected legs from 53 HCs were included in the analysis (Fig. 5).

### Clinical assessment

We investigated the onset region (bulbar, cervical, or lumbosacral), disease duration from onset to clinical assessment, and ALSFRS-R score^37^. Clinical UMN signs included spasticity, brisk deep tendon reflexes, ankle clonus, and Babinski or Chaddock signs. Muscle atrophy, fasciculation, and decreased or absent deep tendon reflexes were regarded as clinical LMN signs. Among 12 items consisting of the ALSFRS-R scoring system, we regarded the sum of the 7th to 9th items as a representative of lower-limb function (denoted as subALSFRS-R hereafter). The neurological examination was performed by a senior specialist in ALS (J-JS).

### Nerve conduction studies

The NCS was conducted by conventional procedures using Nicolet EDX with Viking software (Natus Medical Incorporated, San Carlos, CA, USA). Surface temperatures of lower extremities were maintained over 32˚C. The band-pass filter was set between 3 Hz and 10 kHz. Recording electrodes were placed over the belly of EDB and AH muscles. Reference electrodes were placed 2–3 cm away from the recording electrodes. The peroneal and posterior tibial nerves were stimulated at the midpoint between the medial and lateral malleoli and between the posterior and medial malleolus, respectively. The peak-to-peak amplitudes of CMAPs from the EDB and AH evoked by supramaximal stimulation were recorded. If the NCS recorded at the EDB showed any abnormal response, CMAPs from the TA were additionally obtained by stimulating peroneal nerves below the lateral fibula. A recording electrode was placed over the belly of TA, and a reference electrode was placed 2–3 cm distally. The SI was calculated by CMAP_EDB_/CMAP_AH_ (SI_EDB_) or CMAP_TA_/CMAP_AH_ (SI_TA_).

### Statistical analyses

The Shapiro–Wilk test was used to assess the normality of data. Normally distributed variables were expressed as mean ± standard deviation, and non-normal variables were expressed as median [interquartile range]. A non-parametric Kruskal–Wallis test with Bonferroni correction or Pearson’s Chi-square test was conducted for group comparisons. A two-tailed p < 0.05 was considered statistically significant. All statistical analyses were performed using R 3.6.1 for Windows.

### Ethics approval

This study was approved by the local institutional ethics committee of the Seoul National University Hospital (IRB. No: 2020-0524). Informed consent was waived by the IRB due to the retrospective design and anonymized data. All methods were performed in accordance with the relevant guidelines and regulations.

## Supplementary information

Supplementary file1

## Data Availability

The data that support the findings of this study are available from the corresponding author upon reasonable request.
