# Supplementary material for: Dissociated leg muscle atrophy in amyotrophic lateral sclerosis/motor neuron disease: the ‘split-leg’ sign
Source: Sci Rep. 2020 Sep 24;10:15661. doi: 10.1038/s41598-020-72887-7 (PMC7518279; doi:10.1038/s41598-020-72887-7)

Dissociated leg muscle atrophy in amyotrophic lateral sclerosis/motor neuron disease: the 'split-leg' sign

Young Gi Min[1,a], Seok-Jin Choi[2,a], Yoon-Ho Hong[3], Sung-Min Kim[1], Je-Young Shin[1], Jung-Joon Sung[1]

All supplementary figures were created by YGM using Microsoft PowerPoint (https://products.office.com/en-in/powerpoint version 16.16.3 (181015)).

Supplementary Figure 1. CMAP from 3 muscles according to the diagnosis. For CMAP from all 3 muscles, there was significant reduction in ALS and PMA compared to controls. Notably, in PMA, the reduction was most remarkable for $CMAP_{TA}$ (B) but modest in $CMAP_{AH}$ (C).

*p<0.05, ****p<0.0001.

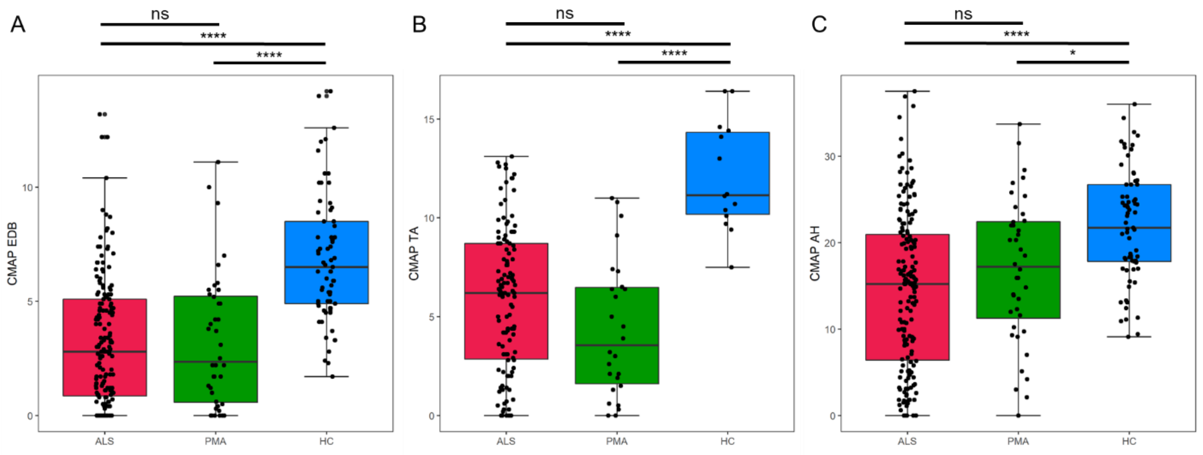

Supplementary Figure 2. Male sex was associated with high SI_{EDB} in healthy controls.

***p<0.001.

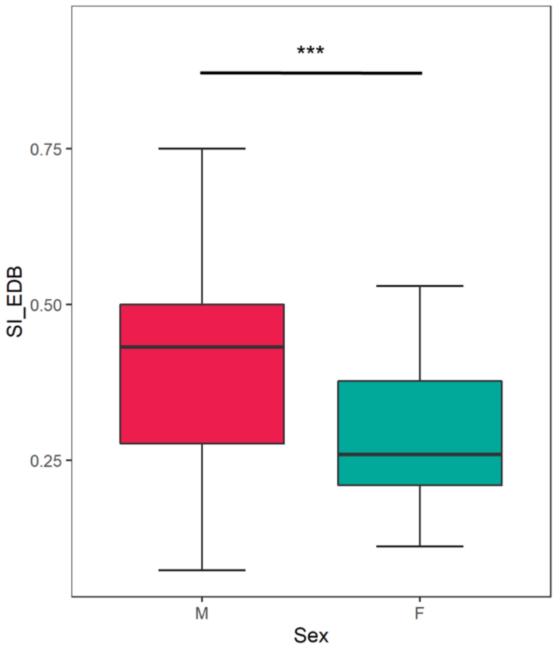

Supplement: Supplementary file 1 — Supplementary file1 [file 41598_2020_72887_MOESM1_ESM.pdf]
